# Social Support Patterns of Middle-Aged and Older Adults Within a Physical Activity App: Secondary Mixed Method Analysis

**DOI:** 10.2196/12496

**Published:** 2019-08-23

**Authors:** Zakkoyya H Lewis, Maria C Swartz, Eloisa Martinez, Elizabeth J Lyons

**Affiliations:** 1 Kinesiology & Health Promotion Department California State Polytechnic University Pomona Pomona, CA United States; 2 Department of Pediatrics-Research The University of Texas MD Anderson Cancer Center Houston, TX United States; 3 Sealy Center on Aging The University of Texas Medical Branch Galveston, TX United States; 4 Department of Nutrition and Metabolism The University of Texas Medical Branch Galveston, TX United States

**Keywords:** social support, aged, middle aged, physical activity, technology, fitness tracker

## Abstract

**Background:**

Physical activity (PA) is critical for maintaining independence and delaying mobility disability in aging adults. However, 27 to 44% of older adults in the United States are meeting the recommended PA level. Activity trackers are proving to be a promising tool to promote PA adherence through activity tracking and enhanced social interaction features. Although social support has been known to be an influential behavior change technique to promote PA, how middle-aged and older adults use the social interaction feature of mobile apps to provide virtual support to promote PA engagement remains mostly underexplored.

**Objective:**

This study aimed to describe the social support patterns of middle-aged and older adults using a mobile app as part of a behavioral PA intervention.

**Methods:**

Data from 35 participants (mean age 61.66 [SD 6] years) in a 12-week, home-based activity intervention were used for this secondary mixed method analysis. Participants were provided with a Jawbone Up24 activity monitor and an Apple iPad Mini installed with the UP app to facilitate self-monitoring and social interaction. All participants were given an anonymous account and encouraged to interact with other participants using the app. Social support features included comments and *likes*. Thematic coding was used to identify the type of social support provided within the UP app and characterize the levels of engagement from users. Participants were categorized as superusers or contributors, and passive participants were categorized as lurkers based on the literature.

**Results:**

Over the 12-week intervention, participants provided a total of 3153 likes and 1759 comments. Most participants (n=25) were contributors, with 4 categorized as superusers and 6 categorized as lurkers. Comments were coded as emotional support, informational support, instrumental support, self-talk, and other, with emotional support being the most prevalent type.

**Conclusions:**

Our cohort of middle-aged and older adults was willing to use the social network feature in an activity app to communicate with anonymous peers. Most of our participants were contributors. In addition, the social support provided through the activity app followed social support constructs. In sum, PA apps are a promising tool for delivering virtual social support to enhance PA engagement and have the potential to make a widespread impact on PA promotion.

**Trial Registration:**

ClinicalTrials.gov NCT01869348; https://clinicaltrials.gov/ct2/show/NCT01869348

## Introduction

### Physical Activity and Social Support

Physical activity (PA) is critical during the aging process to help maintain independence and delay the development of mobility disability. Yet, in a study conducted by Keadle et al in 2016, less than 50% of healthy older adults achieved the recommended PA level [1]. In fact, research has found that adherence to PA guidelines or interventions decreased from 80% to 50% over 12 months [2]. Traditional PA interventions are labor intensive for both the research team and the participants. Recent advances in technology have produced methods (eg, wearable activity trackers) that allow for less labor-intensive PA intervention design [3]. Wearable activity trackers have been shown to be a promising tool to augment traditional PA intervention designs by replacing the need to manually record PA through passive activity tracking [3]. Activity trackers also expand on limited feedback of pedometers by providing goal-setting assistance and extensive feedback on progress and encouraging social interaction [4]. Despite a surge in the use of activity trackers within PA interventions, it is not yet clear how middle-aged and older adults use the virtual support features to promote PA engagement. Given that social support is a psychosocial factor that has been consistently identified as a critical factor for the adoption and maintenance of PA behavior [5,6], it is important for us to examine this influential psychosocial factor to provide insight for future PA intervention designs.

Social support is defined as an interpersonal exchange that increases self-esteem and offers acceptance, value, and motivation to individuals [7]. In a study, individuals who perceived low social support within their social environment were found to be 2 times more likely to be sedentary [8]. Reviews have indicated that shared experience, such as exercising in a group setting (eg, walking club) or being socially connected (eg, community facilities or virtual blogs), can help shape and foster the adoption of PA behavior [9]. Furthermore, empirical evidence has indicated that social support is an influential behavioral change technique that has the potential to encourage PA among older adults [6,10,11] and promote adherence to PA recommendations [5,12]. Using the self-determination theory (SDT) as the framework [13], Vallerand posited that social support can affect the degree of motivation (also referred to as the degree of self-determination) and, in turn, affect health behavior [14]. George et al explored the mechanistic relationship further by using the hierarchical model of intrinsic and extrinsic motivation models and found that perceived social support positively associated with the 3 components of basic psychological needs (autonomy, competence, and relatedness) as indicated by SDT [13,15]. In turn, the psychological needs were positively associated with motivation and PA intention [15]. Thus, we can posit that social support plays a critical role in meeting the basic psychological needs and, in turn, increases motivation to engage in PA.

### Social Support Constructs

Constructs of social support, through social media or conventional means, can be categorized as emotional, informational, instrumental, or appraisal support as described by Heaney and Israel [16]. Emotional support is an expression of empathy, love, trust, or care. Informational support provides advice or information. Instrumental support provides tangible aid. Appraisal support provides information that is useful for self-evaluation [16]. Support can also come from within the individual through self-talk [17,18]. Self-talk can be a discussion with oneself or a multiparty dialog [17], and it can be positive or negative among individuals [18]. With the increase in mobile phone usage and wearable activity tracker usage, individuals seeking support can do so using social networks available in mobile apps. Research shows that social networking sites (SNSs) have been used to provide informational support (eg, guidance), instrumental support (eg, connect individuals with resources), and emotional support. In addition, SNSs have also been used to facilitate behavior change interventions [16,19-22]. Specifically, de la Peña and Quintanilla found that health-related Facebook communities were able to provide informational, instrumental, and emotional support needed for members to achieve their goals [20] through features such as *likes* and *comments* [20]. The *like* feature is a form of emotional support by providing users positive and indirect feedback. The *comments feature* in itself is a form of emotional support. However, it is also used to provide appraisal support (ie, constructive feedback) or instrumental and/or informational support (ie, suggestions or connection with resources).

Nevertheless, individuals vary in how they participate in SNSs to receive and provide social support. Researchers confirmed that the 90-9-1 principle (also known as the 1% rule) developed by digital marketing researchers [23] also reflected the phenomenon observed within the digital health environment [24]. Researchers found that content came within the digital health SNSs or the internet support groups came from superusers and contributors [24-26]. Most content was provided by superusers who represent approximately 1% of the members in the SNSs. Contributors generated a minority of the content, and they represent about 9% of the members. Most members in the digital health support system are considered as lurkers (~90%). These are individuals who observe without active participation [27-29].

### Objective

Although the popularity of receiving and providing social support through SNSs is rising, the usage pattern for health and PA promotion among middle-aged and older adults remains unclear [30]. There is preliminary evidence that older adults who use virtual support provide comments that align within the constructs of social support [31], but they are cited as being apprehensive about communicating with strangers [32] although an increasing number of older adults are on social media sites [33]. Thus, the purpose of this secondary data analysis was to describe the social support patterns among middle-aged and older adults using a mobile phone app as part of a behavioral PA intervention and evaluate them within the constructs of social support. We hypothesize that social support patterns in our cohort of middle-aged and older adults will align with the social support constructs and social network engagement pattern.

## Methods

### Study Design and Population

This study was a secondary mixed-method analysis with data retrieved from a randomized controlled study (Trial Registration: NCT01869348). Data were drawn from a 12-week, randomized controlled behavioral PA study. The primary study’s recruitment and intervention methods are published elsewhere [34]. The eligibility criteria were as follows: (1) aged 55 to 79 years, (2) body mass index of 25 to 35 kg/m^2^, (3) able to read and understand English, (4) able to read the print off of a tablet, and (5) cleared to participate as determined by the Physical Activity Readiness Questionnaire [35]. Participants were enrolled and started the intervention between 2014 and 2016. This secondary analysis included 35 of the 40 study participants who used the UP app (Jawbone, San Francisco, CA) and who always had at least one participant (herein referred to as peers) to communicate with during the week. Due to rolling enrollment, the actual number of peers fluctuated on a weekly basis (between 0 and 10). Of the 40 participants, 5 were not included in this analysis because they either refused to participate in the intervention after the wait-list period or did not have at least one peer to communicate with during any point of the intervention.

### Procedures

Eligible participants were randomized to either the intervention group or the wait-list control group. The intervention provided a wearable activity monitor (UP24 by Jawbone, San Francisco, CA) and a mobile tablet device (iPad Mini by Apple, Cupertino, CA) and received scripted, brief weekly telephone cognitive behavioral counseling. The wait-list control group received all the intervention components after their 12-week final assessment. The UP app was preinstalled on the tablets so that the participants could view their activity and interact with other participants. All participants were given an anonymous account (eg, Monopoly icons) and were *friended* with the other participants and the interventionists. Interventionists used the app for surveillance of the participants only unless there was a software update. The participants were encouraged, but not required, to socialize with others. Participants were instructed to ignore friend requests from unknown users. The app posted individual entries for each person and their peers’ activity progress daily in the *news feed*. Participants were able to comment and *like* the entries; these interactions were analyzed to estimate social support. Interactions were categorized as *received* and *given* support based on whether a comment or *like* was given to or received by a peer. Regardless of their social engagement, participants received notifications from the app when they received a comment or a *like* from a peer. For this reason, *given* support was used for data analysis. Additional UP app features, including leaderboards and challenges, are described in depth elsewhere [36]. The overall study protocol was approved by the university’s institutional review board, and all participants provided written informed consent.

### Data Analysis

NVivo 11 Pro (QSR International) was used for qualitative analysis, and SPSS version 20 (IBM, Armonk, NK) was used for quantitative analysis. Descriptive analyses were conducted using means and frequencies for comments given and received. Furthermore, interquartile range (IQR) for given support (comments or *likes* given to peers) was used to identify superusers and lurkers because it approximated the participants’ engagement with social features. Participants above the 75th percentile in both social support categories (given *likes* and comments) were classified as superusers. Participants below the 25th percentile in both social support categories were classified as lurkers. For qualitative analysis, a combination of directed and conventional qualitative content analysis was used to analyze the app comments [37]. Codes were based on major social support constructs: emotional support, informational support, instrumental support, appraisal support, and self-talk [10,13,14]. Additional codes were developed through conventional qualitative analysis from reading through the comments. Moreover, 2 graduate-level investigators independently coded the comments, and agreement was determined using the NVivo software. Disagreement was settled through discussion and joint review of the comments among coders and the principal investigator (EL) who is a behavioral scientist. Due to protocol restrictions, no quotes from participants were abstracted from the app. Only the quantitative report and the coded qualitative themes are reported.

## Results

### Descriptive Information

Table 1 displays the baseline characteristics of the participants (n=35). On average, participants had 8 peers (range 4-13) to socialize with over 12 weeks.

Throughout the study, there were 3153 *likes* and 1759 comments. The most *likes* received by one person was 524, and the most comments were 291 with a median of 61 *likes* and 32 comments received. Of 35 participants, 3 did not receive a *like* or comment over the 12-week period. The median number of *likes* given was 2, with a range of 0 to 986 and an IQR of 40. The median number of comments given was 14, with a range of 0 to 344 and an IQR of 45. The median number of self-talk comments given was 4, with a range of 0 to 232 and an IQR of 16. Moreover, 11% participants (4/35) were above the 75th percentile in given *likes* and comments. These 4 superusers combined accounted for 72.60% (2289/3153) and 51.28% (902/1759) of the total *likes* and comments, respectively. Conversely, 17% participants (6/35) can be classified as lurkers because they were below the 25th percentile of comments given, and they did not give any *likes*. The remaining 25 participants were classified as contributors—the *likes* or comments given were within the IQR. This group accounted for 27.40% (864/3153) *likes* and 48.72% (857/1759) comments. Lurkers had fewer peers (average: 6; range: 4-9) to communicate with throughout the 12 weeks than contributors (average: 8; range: 6-13) and superusers (average: 9; range: 8-11). Complete social support values for all participants are depicted in Table 2. The *likes* and comments in the table reflect given support, as this reflects the participant’s engagement with the social features.

**Table 1:** Baseline demographic characteristics by study group (N=35).

Characteristics		Intervention (n=19)	Wait-list control (n=16)	Total (N=35)	
Female, n (%)		16 (84)	13 (81)	19 (83)	
**Ethnicity, n (%)**					
	Non-Hispanic white	11 (58)	9 (56)	20 (57)
	Other	8 (42)	7 (44)	15 (43)
College graduate, n (%)		12 (63)	10 (63)	22 (63)	
Age, mean (SD)		61.32 (5)	62.06 (7)	61.66 (6)	
Body mass index, kg/m^2^, mean (SD)		29.99 (3)	30.80 (4)	30.36 (3)	

### Common Themes

**Figure 1:**
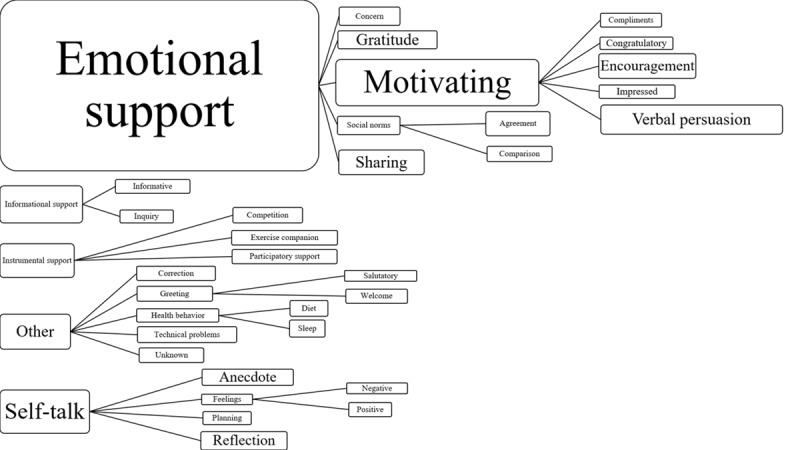
Social support themes. The size of each box represents the prevalence of the different comment themes (not to scale). Study themes were developed based on the work of Heaney and Israel, Cavallo et al, and Cousins et al.

The comments within the app mostly followed the major social support constructs, as described in the Introduction section [10,13,14]. The only theme that was not prevalent was appraisal support. Some comments that were useful for self-evaluation were coded as a subtheme of emotional support. In addition to emotional support, other major themes included informational support, instrumental support, self-talk, and *other* theme. Each major theme had additional subthemes. Figure 1 illustrates the hierarchy of major and subthemes in the comments.

The intervention group participants gave more comments than the wait-list control group participants, but the most prevalent themes were the same between the 2 groups. Agreement between the 2 coders ranged from 53.4% to 99.4% for each theme. The lowest agreement was with self-talk (67.9%) and emotional support (53.44%). Table 3 displays the number of comments given by the participants per major theme. Several comments were coded into numerous themes. Emotional support was the most prevalent, followed by self-talk, other themes, informational support, and instrumental support.

Emotional support was further categorized as concern, gratitude, sharing, motivating, and social norms. *Concern* comments were those that expressed concern for their peer’s health and well-being. *Gratitude* comments expressed thanks to fellow peers for their support. *Sharing* comments were conversation-like posts. *Motivating* comments were further categorized as congratulatory, encouragement, impressed, compliment, and verbal persuasion. *Verbal persuasion* were short, encouraging comments such as *woo-hoo* and *yay*. *Social norms* was further categorized as agreement and comparison.

Subthemes of self-talk included anecdote, feelings (positive and negative), planning, and reflection. Anecdotes were comments that shared personal information or a personal story but were not directed to a peer. *Positive* or *negative* comments toward an individual’s own activity were coded as *feelings*. There were no negative comments between peers. *Planning* comments were the result of an individual planning future PA. Comments where an individual would reflect on their past PA or other health behaviors were coded as *reflection*.

Other themes were subcategorized as correction, technical problems, greeting, health behavior, and unknown. Users cannot edit a previous comment within the app; therefore, additional comments that fixed a previous comment were coded as a *correction*. Comments that expressed technical issues with the Up24 band or the app were coded as *technical problems*. *Greeting* comments were further subcategorized as welcome and salutation. Users had the option to also monitor their sleep and diet behavior, which were the 2 subthemes for *health behavior*. Finally, any other comments that could not be coded into the aforementioned themes were coded as *unknown*.

Informational support included informative and inquiry comments. Informative comments educated peers on PA, the app, or the Up24 band, and inquiry comments posed a question to peers. Instrumental support was further categorized as competition, exercise companion, and participatory support. Comments that mentioned an *exercise companion* differed from *participatory support* because the exercise companion was exercising with the individual’s friend or family member, whereas participatory support came from discussing meeting for in-person exercise with their peers in the study. Participants were not expected to exercise with one another, but comments indicated that participants contacted one another and walked together on at least 12 occasions. All participatory support was organized in the app among the participants.

**Table 2:** Participants’ characteristics and social engagement (N=35; 1-16 were wait-list control participants, and 17-35 were intervention participants).

No	Gender	Peers	*Likes* (N=3153), n (%)	Comments (N=1759), n (%)	Self-talk comments (N=758), n (%)
1^a^	F^b^	10	299 (9.48)	166 (9.43)	31 (4.1)
2^a^	F	9	986 (31.27)	340 (19.32)	105 (13.9)
3^a^	M^c^	7	544 (17.25)	344 (19.55)	232 (30.6)
4^d^	M	6	2 (0.06)	0 (0.00)	0 (0.0)
5^d^	F	8	0 (0.00)	16 (0.90)	13 (1.7)
6^d^	F	8	0 (0.00)	4 (0.22)	2 (0.3)
7^d^	F	13	2 (0.06)	9 (0.51)	3 (0.4)
8^d^	M	10	1 (0.03)	5 (0.28)	3 (0.4)
9^d^	F	9	45 (1.42)	30 (1.70)	18 (2.4)
10^d^	F	9	0 (0.00)	1 (0.05)	1 (0.1)
11^d^	F	8	6 (0.19)	5 (0.28)	4 (0.5)
12^e^	F	7	0 (0.00)	0 (0.00)	1 (0.1)
13^3^	F	8	0 (0.00)	0 (0.00)	0 (0.0)
14^e^	F	9	0 (0.00)	0 (0.00)	0 (0.0)
15^e^	F	4	0 (0.00)	0 (0.00)	0 (0.0)
16^e^	F	6	0 (0.00)	0 (0.00)	0 (0.0)
17^a^	F	11	460 (14.58)	52 (2.95)	26 (3.4)
18^d^	F	6	5 (0.15)	57 (3.24)	16 (2.1)
19^d^	F	6	24 (0.76)	223 (12.67)	82 (11.2)
20^d^	M	7	0 (0.00)	0 (0.00)	0 (0.0)
21^d^	F	6	2 (0.06)	194 (11.0)	85 (11.2)
22^d^	F	6	5 (0.15)	40 (2.27)	14 (1.8)
23^d^	F	6	8 (0.25)	15 (0.85)	8 (1.1)
24^d^	F	11	0 (0.00)	76 (4.32)	38 (5.0)
25^d^	M	8	1 (0.03)	23 (1.30)	2 (0.3)
26^d^	F	6	338 (10.7)	14 (0.79)	9 (1.2)
27^d^	F	6	13 (0.41)	17 (0.96)	12 (1.6)
28^d^	F	6	26 (0.82)	13 (0.73)	4 (0.5)
29^d^	F	6	0 (0.00)	24 (1.36)	16 (2.1)
30^d^	F	8	279 (8.84)	46 (2.61)	17 (2.2)
31^d^	M	10	1 (0.03)	1 (0.05)	1 (0.1)
32^d^	F	13	40 (1.26)	4 (0.22)	0 (0.0)
33^d^	F	12	0 (0.00)	1 (0.05)	1 (0.1)
34^d^	F	10	66 (2.09)	34 (1.93)	14 (1.8)
35^a^	F	6	0 (0.00)	0 (0.00)	0 (0.0)

^a^Superuser.

^b^F: female.

^c^M: male.

^d^Contributor.

^e^Lurker.

**Table 3:** Number of comments by themes.

Themes			Intervention (n=19 participants)	Wait-list controls (n=16 participants)	Total participants (N=35)		
**Emotional support^a^**, **n comments (%)**			**475 (50.6)**	**596 (67.3)**	**1071 (58.72)**		
	Concern^b^		29 (3)	24 (3)	53 (3)	
	Gratitude^b^		75 (8)	107 (12.1)	182 (10.0)	
	**Motivating^b^**		**317 (33.8)**	**420 (47.4)**	**737 (40.4)**	
		Compliment^c^	41 (4)	54 (6)	95 (5)
		Congratulatory^c^	53 (6)	60 (7)	113 (6.2)
		Encouragement^c^	75 (8)	89 (10)	164 (9.0)
		Impressed^c^	45 (5)	70 (8)	115 (6.3)
		Verbal persuasion^c^	155 (16.5)	215 (24.3)	370 (20.3)
	Sharing^b^		92 (10)	80 (9)	172 (9.4)	
	**Social norms^b^**		**42 (5)**	**18 (2)**	**60 (3)**	
		Agreement^c^	13 (1)	5 (1)	18 (1)
		Comparison^c^	32 (3)	13 (2)	45 (3)
**Informational support^a^, n (%)**			**64 (7)**	**18 (2)**	**82 (5)**		
	Informative^b^		23 (2)	2 (0)	25 (1)	
	Inquiry^b^		40 (4)	17 (2)	57 (3)	
**Instrumental support^a^, n (%)**			**48 (5)**	**11 (1)**	**59 (3)**		
	Competition^b^		20 (2)	0 (0)	20 (1)	
	Exercise companion^b^		17 (2)	10 (1)	27 (2)	
	Participatory support^b^		12 (1)	1 (0)	13 (1)	
**Self-talk^a^, n (%)**			**220 (23.4)**	**188 (21.2)**	**408 (22.4)**		
	Anecdote^b^		83 (9)	85 (10)	168 (9.2)	
	**Feelings^b^**		**49 (5)**	**38 (4)**	**87 (5)**	
		Negative^c^	15 (2)	6 (1)	21 (1)
		Positive^c^	34 (4)	31 (4)	65 (4)
	Planning^b^		42 (5)	16 (2)	58 (3)	
	Reflection^b^		98 (10)	78 (9.0)	176 (9.6)	
**Other^a^, n (%)**			**132 (14.1)**	**72 (8)**	**204 (11.2)**		
	Correction^b^		6 (1)	9 (1)	15 (1)	
	**Greeting^b^**		**23 (2)**	**24 (3)**	**47 (3)**	
		Salutatory^c^	10 (1)	11 (1)	21 (1)
		Welcome^c^	15 (2)	10 (1)	25 (1)
	**Health behaviors^b^**		**52 (6)**	**22 (3)**	**74 (4)**	
		Diet^c^	7 (1)	0 (0)	7 (0)
		Sleep^c^	46 (5)	23 (3)	69 (4)
	Technical problems^b^		33 (4)	16 (2)	49 (3)	
	Unknown^b^		6 (1)	3 (0)	9 (1)	
Total comments^d^			939 (51.5)	885 (48.5)	1824	

^a^Major themes; themes were developed based on the work of Heaney and Israel, Cavallo et al, and Cousins et al.

^b^**S**ubmajor themes.

^c^Minor themes.

^d^Some comments were coded into several submajor or minor themes. Therefore, the total depicts the total number of comments under the major theme.

## Discussion

### Principal Findings

Our exploratory study on social support patterns of middle-aged and older adults using a mobile app found that without being mandated to socialize with other participants, the 35 participants who used the app as part of the intervention produced a total of 1759 comments and 3153 *likes* over a 12-week intervention period. Of 35 participants, 4 were classified as superusers because they were above the 75th percentile for all support categories (given *likes* and comments), whereas 6 participants were classified as lurkers for falling below the 25th percentile in given comments and did not give any *likes*. Common themes coded from the content of comments followed constructs of social support, with the most prevalent comments classified as emotional support followed by self-talk.

Our evaluation partially supports the 90-9-1 principle in that the smallest portion of participants was superusers [24]; however, our sample did not follow the same distribution. We found that 17% (n=6) of participants were lurkers, 71% (n=25) were contributors, and 11% (n=4) were superusers. Contributors were the largest group, which is contrary to the 90-9-1 principle that states lurkers are the most prevalent [24]. Despite the increase in superusers and contributors, their contribution in the app was similar to previous evaluations. Van Mierlo investigated the 90-9-1 principle in 4 digital health social networks and found that the superusers, the top 1%, accounted for 73.6% of posts, whereas contributors accounted for 24.7% of posts [24]. In our study, superusers and contributors accounted for a comparable 72.6% and 27.1% of *likes*, respectively. The larger proportion of superusers and contributors in our sample may be the result of the intimate nature and anonymity of the study. At any given time during the study, there were only 1 to 10 peers for a participant to interact with versus the possible hundreds of peers on an SNS. It is unclear whether the size of the social group may have affected support provision behavior. However, too few peers may have inhibited social interaction and is a clear limitation of this exploratory investigation.

Previous research suggests that older adults are apprehensive to communicate with strangers [32]; the anonymous nature of the team may have also contributed to increased interactions in our study, as individuals were known by their icon rather than their real name. Some participants used these icons as a conversation starter, which helped to increase social engagement. This may have also affected the type of support that was provided. The anonymity did not result in negative comments toward peers, only in the form of self-talk. Participants may have been supportive because of their shared interests in the intervention or the surveillance of the intervention. Future research should investigate the effect of group size, anonymity, and icon personae on social interaction within apps or SNSs.

Although the number of comments varied between intervention and wait-list control participants, the most prevalent themes remained the same. The rank of themes by prevalence was emotional support, self-talk, other, informational support, and instrumental support. This trend is similar to that observed in women who used Fitbit and its Web-based social network [38]. A total of 20 women enrolled in a 6-week study were given a Fitbit Flex to monitor their activity and access to the Web-based Fitbit system. The social features of the Fitbit system included a message board for communication and a leaderboard [38]. The study found the most prevalent comments were motivational (emotional support), followed by sharing of PA ideas (informational support) and exercising with others (instrumental support). Self-talk and other types of comments were not reported. Results of the multilevel model analyses showed that social contact had a significant effect on PA, but it is unclear which type of support influenced PA the most [38].

Similar results were found in a PA intervention for individuals with Parkinson disease. Participants were given a Fitbit zip and assigned a peer mentor to help promote PA. The peer mentors were *friends* with the participants through the Fitbit system. As *friends*, the mentors were able to provide emotional support, through comments and *likes*, and instrumental support, through social comparison of activity. After an 8-week period of peer mentorship, participants increased their PA by 31% [39]. Yet, it is still unclear which type of support was driving the PA change. In traditional in-person social networks, emotional support has a considerable impact on PA compared with other types of support [40,41]. More research is needed to determine how PA is affected by each type of support (emotional support, instrumental support, informational support, and self-talk) within a virtual environment. Studies by Colon-Semenza et al and Arigo mandated social support, whereas this study did not [38,39]; researchers should also investigate the impact of mandated versus organic social engagement.

### Limitations

The informed consent document did not ask for clearance to share comments from the app, as we did not expect virtual social support to be so prevalent. Furthermore, challenges related to the recruitment strategy of pilot trial should be considered in interpreting these data, as they caused the number of simultaneous peers to shift throughout the intervention period. Due to these limitations, this study is limited to a description and was not able to further examine the comments or their potential impacts on intervention effectiveness. The intervention was designed to impact PA behavior, which provides further limitations for this secondary data analysis. Participants’ prior experience on social media use was not captured. As the inclusion of wait-list participants, change in PA as a result of social interaction could not be assessed. Therefore, our results are exploratory in nature, and no conclusions on the relationship of social support and PA could be made. Preliminary results from Arigo [38] and Colon-Semenza et al [39] suggest that more virtual social interaction results in more PA, but these studies were among young adults and Parkinson disease patients, respectively. These results are not necessarily generalizable to middle-aged and older adults. Furthermore, this study neither provides a network analysis of the relationship between lurkers, contributors, and superusers nor accounts for the fluctuating number of peers. This clear limitation of this study should be addressed in future trials by ensuring timely recruitment in small cohorts. Our thematic coding of the participants’ comments was conducted independently by 2 coders following qualitative analysis guidelines, but an external researcher was not involved in the study to review the themes. This may limit the internal validity of our evaluation. Most of our participants were non-Hispanic white and female, and future research should include a more diverse sample. The strength of this study includes a thorough description of how older adults support their unknown peers using an app and evidence of the acceptability of anonymous social support in addition to counseling calls from research staff.

### Conclusions

Use of wearable activity monitors that have a social networking feature is on the rise both commercially and in research [38,42]. Their features, similar to other SNSs, have the potential to make a widespread impact on PA promotion in the clinical and community settings. However, to our knowledge, use of the social networking features of these devices to provide social support are seldom reported or evaluated [30]. The results of our study suggest that middle-aged and older adults were willing to use social tools in a PA app to communicate with unknown, anonymous peers (total of 3153 *likes* and 1759 comments over 12 weeks). Social support in our study also happened organically without being required as a part of an intervention. Social support provided in the app followed constructs of social support [37]. The most prevalent type of support was emotional support. Contrary to the 90-9-1 principle, most participants were contributors (71.4%), with only 11.4% superusers and 17.1% lurkers. In combination with the other implemented behavioral change techniques [4], our findings provide further support for the potential usefulness of wearable activity monitors as a promising intervention tool to encourage behavior change. Future research is needed to investigate the potential of these social support features to change PA behavior. Practitioners should be aware that these features exist in many available PA apps and may be used by patients to provide and receive support. However, education needs to be provided regarding information security.

## Abbreviations

IQR: interquartile range
PA: physical activity
SDT: self-determination theory
SNS: social networking site
